# Growth modulation response in vertebral body tethering depends primarily on magnitude of concave vertebral body growth

**DOI:** 10.1007/s43390-024-00909-9

**Published:** 2024-06-04

**Authors:** Craig R. Louer, Vidyadhar V. Upasani, Jennifer K. Hurry, Hui Nian, Christine L. Farnsworth, Peter O. Newton, Stefan Parent, Ron El-Hawary, Pediatric Spine Study Group (PSSG)

**Affiliations:** 1 Department of Orthopedic Surgery, Vanderbilt University Medical Center, 2200 Childrens Way, Suite 4202, Nashville, TN 37212 USA; 2 Department of Orthopedics, Rady Children’s Hospital, San Diego, CA USA; 3 Department of Orthopaedic Surgery, University of California San Diego, San Diego, CA USA; 4 Division of Orthopaedic Surgery, IWK Health Centre, Halifax, NS Canada; 5 Department of Biostatistics, Vanderbilt University Medical Center, Nashville, TN USA; 6 Department of Surgery, Faculty of Medicine, Universite´ de Montre´Al, Montre´Al, QC Canada

**Keywords:** Growth modulation, Vertebral body tethering, Scoliosis, 3D, Remodeling

## Abstract

**Purpose:**

There is variability in clinical outcomes with vertebral body tethering (VBT) partly due to a limited understanding of the growth modulation (GM) response. We used the largest sample of patients with 3D spine reconstructions to characterize the vertebra and disc morphologic changes that accompany growth modulation during the first two years following VBT.

**Methods:**

A multicenter registry was used to identify idiopathic scoliosis patients who underwent VBT with 2 years of follow-up. Calibrated biplanar X-rays obtained at longitudinal timepoints underwent 3D reconstruction to obtain precision morphological measurements. GM was defined as change in instrumented coronal angulation from post-op to 2-years.

**Results:**

Fifty patients (mean age: 12.5 ± 1.3yrs) were analyzed over a mean of 27.7 months. GM was positively correlated with concave vertebra height growth (*r* = 0.57, *p* < 0.001), 3D spine length growth (*r* = 0.36, *p* = 0.008), and decreased convex disc height (*r* = − 0.42, p = 0.002). High modulators (patients experiencing GM > 10°) experienced an additional 1.6 mm (229% increase) of mean concave vertebra growth during study period compared to the Poor Modulators (GM < − 10°) group, (2.3 vs. 0.7 mm, *p* = 0.039), while convex vertebra height growth was similar (1.3 vs. 1.4 mm, *p* = 0.91).

**Conclusion:**

When successful, VBT enables asymmetric vertebra body growth, leading to continued postoperative coronal angulation correction (GM). A strong GM response is correlated with concave vertebral body height growth and overall instrumented spine growth. A poor GM response is associated with an increase in convex disc height (suspected tether rupture). Future studies will investigate the patient and technique-specific factors that influence increased growth remodeling.

## Introduction

Vertebral Body Tethering (VBT) is an emerging procedure in the treatment of Adolescent Idiopathic Scoliosis (AIS) which differs from the current surgical standard (spine fusion) by preserving some degree of spine flexibility [1, 2]. Surgical application of a strong, flexible, tether to a scoliotic convexity induces immediate curve correction. Ideally, continued correction (termed “growth modulation” (GM)) will occur as determined by the Hueter-Volkmann principle due to the patient’s continued growth and the altered biomechanical forces from VBT tensioning. Widespread acceptance of this procedure has been limited by its varied outcomes and higher risk of re-operation (10–30%) [3–6] due to under- or over-correction.

Limited understanding of the mechanism and determinants of growth modulation represents a key knowledge gap in our current application of this technique. Elucidating the morphologic changes of spine segments that accompany GM are essential to this understanding, as are the patient-specific and technical factors that influence the intensity of the GM response. This study focuses on the former question of morphologic changes in the spine segments. We hypothesize that VBT induces changes in the vertebral body and disc morphology that determine the degree of GM. In this study, we use the largest set published to date of three-dimensional (3D) reconstructions to characterize the changes in disc and vertebra morphology that accompany growth modulation during the first two years following VBT.

## Materials and methods

This is a retrospective study of radiographic and clinical data from a multicenter pediatric spine registry (Pediatric Spine Study Group). Patient data and radiographs were de-identified prior to sharing with the primary institution. Study protocols were approved by the local Institutional Review Board. Inclusion criteria included juvenile or adolescent patients with idiopathic scoliosis who were treated with VBT at least 2 years prior to study initiation. Patients were excluded if they had prior spine surgery or underwent surgical revision prior to the 2-year follow-up time point. Age, sex, race, weight, height, BMI, and surgical dates/revisions were obtained from the registry. Markers of skeletal maturity such as Risser sign, triradiate cartilage status, and proximal femur maturity index (PFMI) [7] were determined from the pre-op anteroposterior radiograph by a single reviewer (C.R.L.). Tether ruptures were determined using the criterion of > 5° increase in screw angle at any level between the first post-op and 2-year post-op radiographs.

**Fig. 1:**
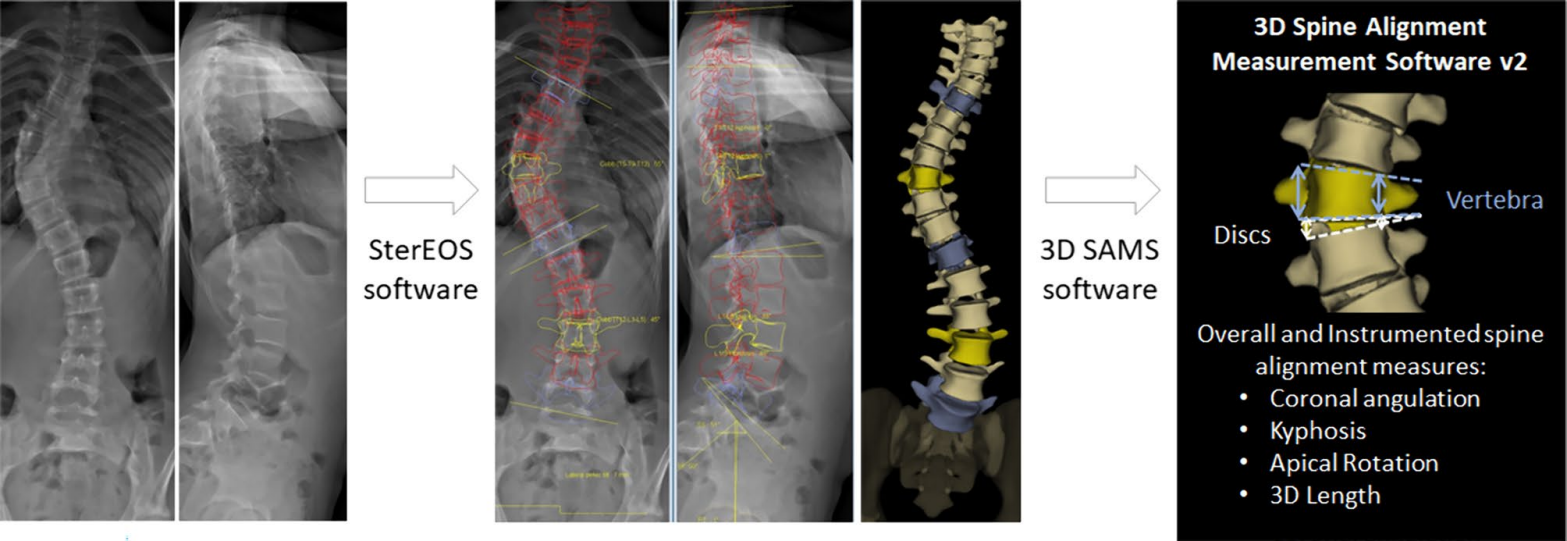
Method of 3D spine reconstructions and morphologic measurements

De-identified, calibrated biplanar radiographs (EOS imaging, Paris FR) were obtained from registry participants and underwent 3D reconstruction by a single research engineer (J.K.H) using sterEOS software (EOS imaging, SA, Paris France). Computer-assisted fitting of 3D ellipses to represent the vertebra endplates has been previously validated compared to cadaveric, and mathematically generated models [8], in addition to CT imaging [9]. Morphologic measurements of individual vertebra and discs, as well as overall instrumented spine alignment was attained through a custom MATLAB (MathWorks, Natick, MA) script (3D SAMS (Spinal Alignment Measurement Software) software v2, Rady Children’s Hospital, Orthopedic Surgery Division, San Diego, CA) using previously described methods (Fig. 1) [10].

**Fig. 2:**
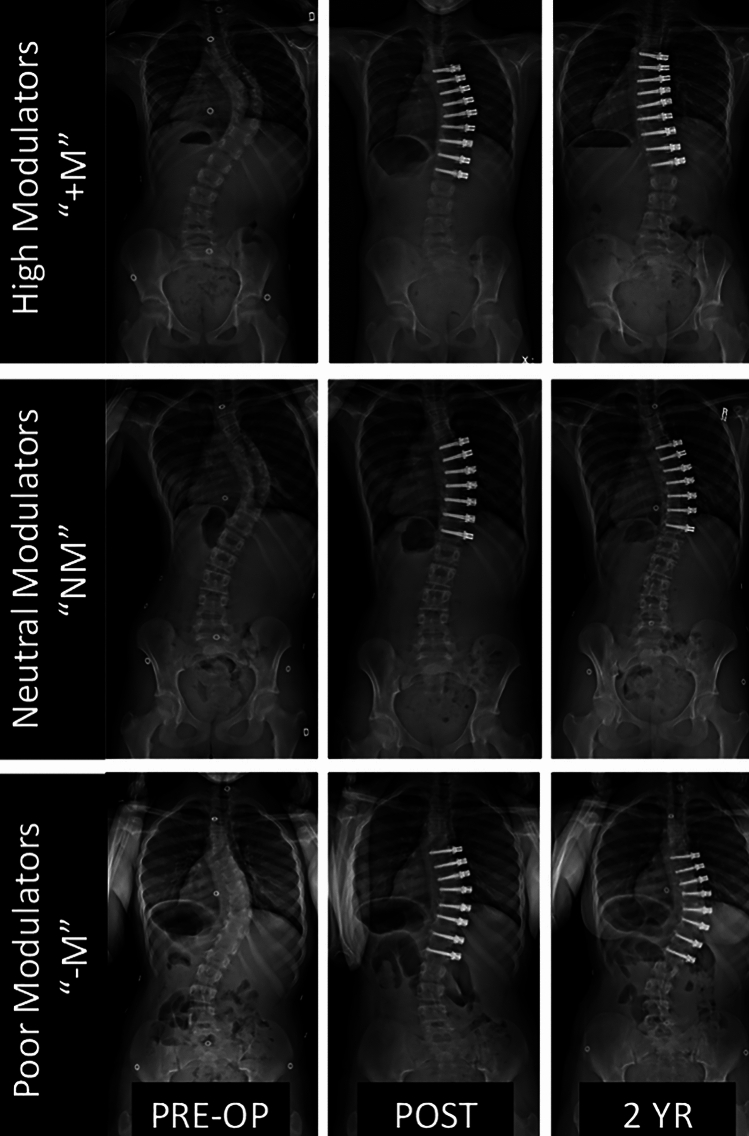
Examples of patients in each of our GM groups (High, Neutral, and Poor Modulators) demonstrating the wide variety in clinical outcomes with VBT

Overall 3D changes of instrumented spine alignment were compared using the Wilcoxon signed rank test. A right-sided curve was represented by a negative coronal angulation value, such that an increase in angulation value represents an improvement in the deformity. Spearman’s correlation was performed between the magnitude of GM (2-year minus post-op coronal angulation) and morphologic change in vertebrae and discs. Additionally, we chose to perform a sub analysis at each patient’s apex, where tether tension and effect is more uniform. For this analysis, we limited our levels to the three apical vertebra and two apical discs. The cohort was also divided into high modulators (“ + M”; GM > 10°), Neutral Modulators (“NM”; -10° < GM < 10°), and poor modulators (“–M”; GM < − 10°; Fig. 2) with the goal of identifying morphologic differences between successful and unsuccessful cases of GM.). Between-group comparison was performed using the Kruskall–Wallis test, followed by post-hoc Dunn’s test with Holm adjustment. Two-sided *p*-values of < 0.05 were considered statistically significant. We deliberately avoid comparing the demographics of these subgroups for two reasons: (1) univariable analysis often gives misleading results on association due to correlations between predictors and (2) it does not make sense to draw inference for an outcome’s effect on demographics. To examine the effect of multiple factors, a multivariate model is needed. Statistical analysis was performed using R 4.1.0 software (Vienna, Austria).

## Results

**Fig. 3:**
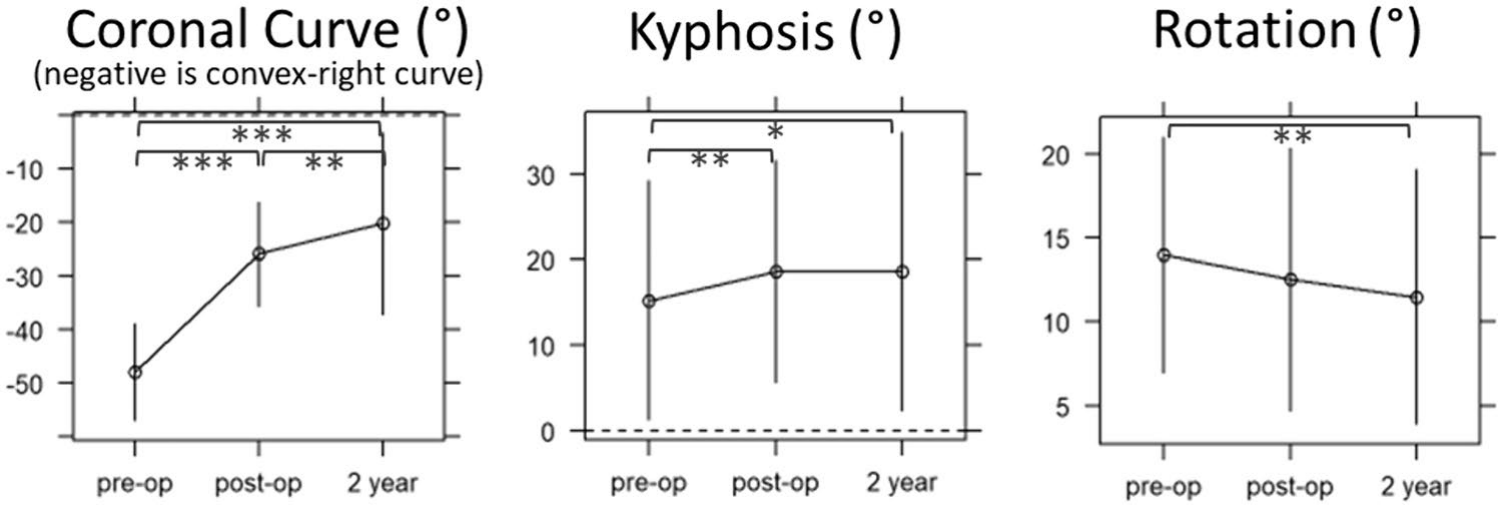
3D changes in overall instrumented spine alignment following VBT. Mean ± SD. Wilcoxon signed rank test: *0.05 < *p* < 0.01, **0.01 < *p* < 0.001, ***p < 0.001

138 patients met the inclusion criteria. Three were excluded due to revision procedure and 85 did not have available biplanar imaging at all timepoints or had errors with 3D processing. The final cohort consisted of 50 patients (mean age: 12.5 ± 1.3 years; Table 1). An average of 44.1 days elapsed from pre-op radiograph to surgery, with another 42.2 days elapsing between surgery and post-op evaluation. Average follow-up was 27.7 months from the pre-op visit and 24.9 months from the post-op visit. There were 16 tether ruptures detected in this cohort from 14 unique patients (28%). Only two ruptures occurred at peri-apical disc spaces. Considering the cohort as a whole, 3D alignment changes within the instrumented curve showed improvement in coronal angulation with a wide distribution of outcomes at 2-years (Fig. 3). There was a minimal increase in 3D kyphosis and minimal decrease in apical rotation within the instrumented spine after two years.

**Table 1:** Demographics and baseline data for Idiopathic Scoliosis patients receiving VBT

Variable	N	
Age (years)	50	12.5 ± 1.3
Sex	50	
Female		92% (46)
Male		8% (4)
Race (database-reported)	50	
White		92% (46)
Black		4% (2)
Other		4% (2)
Weight (kg)	50	43.2 ± 9.4
Height (cm)	50	153.6 ± 8.3
BMI (kg/m^2^)	50	18.2 ± 2.9
Triradiate cartilage status	49	
Open		41% (20)
Closed		59% (29)
Risser Sign	49	
0		71% (35)
1		12% (6)
2		10% (5)
3		2% (1)
4		4% (2)
Proximal femur maturity Index (PFMI)	49	
2		16% (8)
3		39% (19)
4		43% (21)
5		2% (1)
Instrumented cobb (“- “ is R-sided curve)	50	-48.5 ± 9.0
Instrumented kyphosis	50	16.3 ± 13.5
Apical vertebra rotation	50	13.9 ± 7.2

x ± s represents mean ± SD. N is the number of non-missing values. Numbers after proportions are frequencies

On a patient level, improvement in instrumented coronal angulation from post-op to 2-years was positively correlated with concave vertebra height growth (*r* = 0.57, p < 0.001), 3D spine length growth (*r* = 0.36, *p* = 0.008), and decrease in convex disc height (*r* = − 0.42, *p* = 0.002; Table 2).

**Table 2:** Correlations of GM magnitude with morphologic changes of spine from post-op to 2-years following VBT

	Concave vertebrae change	Convex vertebrae change	Concave disc change	Convex disc change	3D spine change
GM Magnitude Spearman’s correlation coefficient (r)	0.565	0.185	0.192	− 0.421	0.361
*p*-value	< 0.001	0.184	0.169	0.002	0.008

**Fig. 4:**
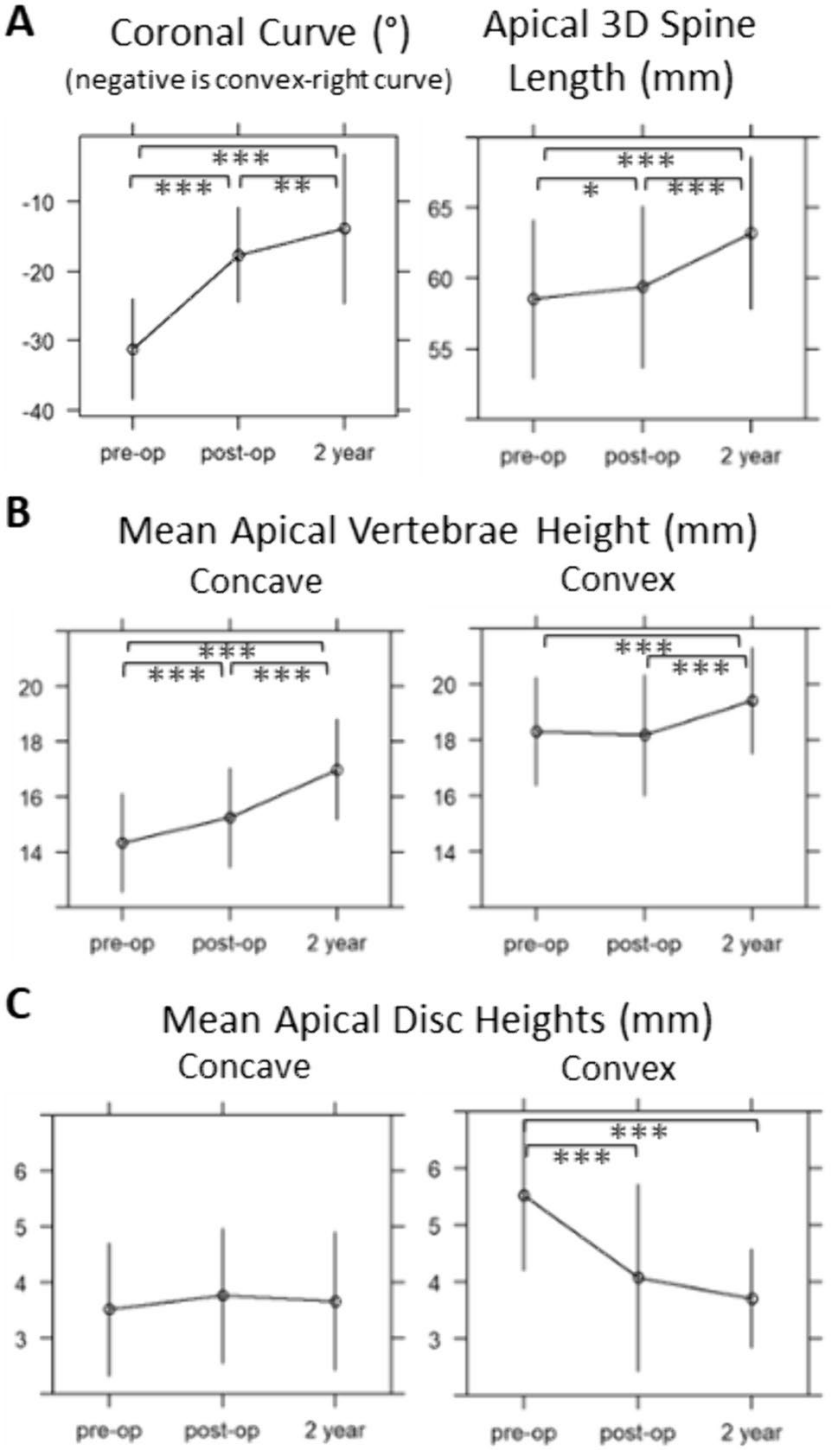
Morphologic changes of the curve apex following VBT, whole cohort. Mean ± SD. Wilcoxon signed rank test: *0.05 < *p* < 0.01, **0.01 < *p* < 0.001, ***p < 0.001. Apical vertebra grew asymmetrically from pre-op to 2-year (mean concave growth 2.5 mm [95% CI: 2.2–2.9 mm] vs. convex 1.1 mm [0.7–1.5 mm]). From post-op to 2-year, apical vertebra mean concave growth was 1.7 mm [1.2–2.1 mm] while convex growth averaged 1.3 mm [0.9–1.6 mm]. Apical disc heights did not significantly change during the two years following tether placement

Tether placement results in morphologic changes at the apex from pre-op to post-op evaluations (Fig. 4). The apical convex discs lose height (mean − 1.4 mm; *p* < 0.001) and concave vertebrae grow (+ 0.9 mm; *p* < 0.001) between pre-op and post-op first-erect Xray. It is notable that an average of 86.3 days elapsed between pre-op and post-op evaluations with + 0.9 mm apical spine length over that time. On average, apical coronal angulation improved by 13.6° (*p* < 0.001) with tether placement and continued to improve by 3.8° (*p* = 0.004) in the subsequent two years. Apical vertebra grew asymmetrically from pre-op to 2-year (mean concave growth 2.5 mm vs. convex 1.1 mm).

**Fig. 5:**
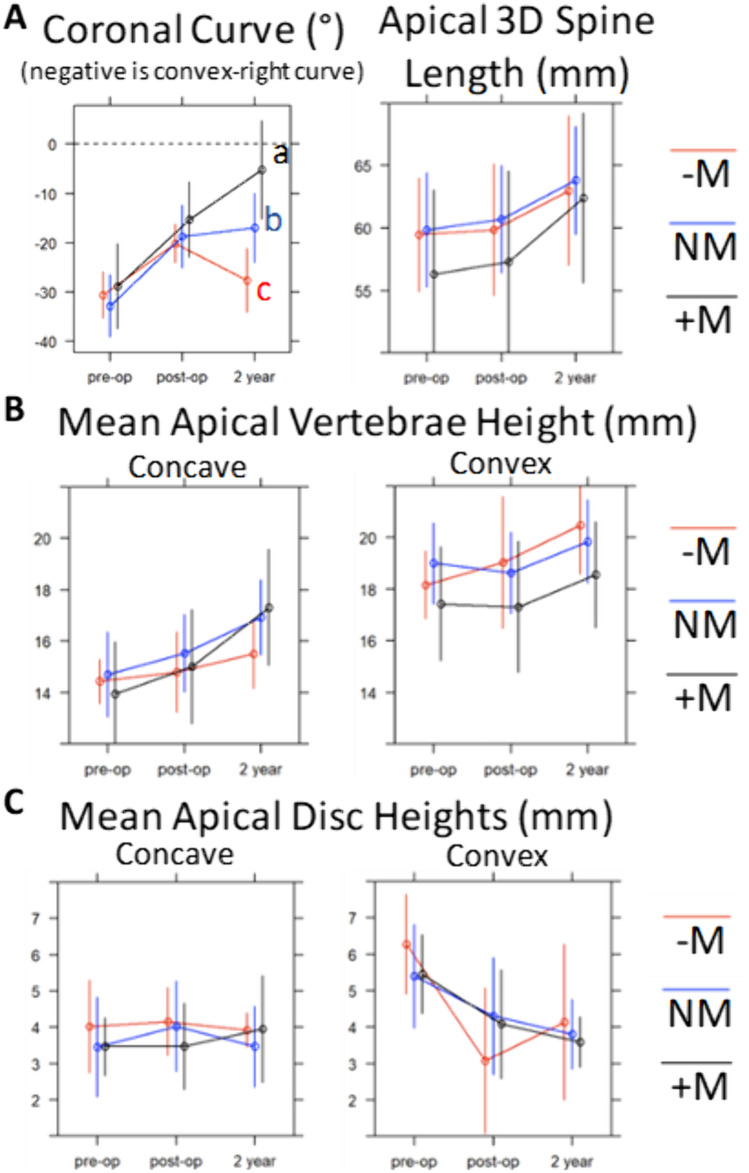
Morphologic changes of the curve apex following VBT: by growth modulation group (“ + M” = High modulator, “NM” = Neutral modulator, “-M” = Poor modulator). Mean ± SD. Between-group comparison at each timepoint performed with two-sample t-test with Dunn post-hoc pairwise comparison (adjusted): a,b,c represent statistical difference (*p* < 0.05) among modulation groups at a given timepoint

When categorized by the magnitude of GM response, 18/50 (36%) patients were high modulators (“ + M”; GM > 10°), 27 (54%) of patients were neutral modulators (“NM”; − 10° < GM < 10°), and 5 (10%) were poor modulators (“-M”; GM < -10°). Pre-op and first post-operative apical coronal angulation and morphologic measures were statistically similar between groups (Fig. 5). After two years, adjusted pairwise comparison showed only a coronal angulation difference between all three groups: + M vs. NM (− 5.2° vs. − 17.0°; p = 0.002), + M vs. –M (− 5.2° vs. − 27.6°; *p* = 0.0002), and NM vs. –M (− 17.0° vs. − 27.6°; *p* = 0.029). There were no other statistical differences among the groups when looking at individual timepoints, however, there are visual trends that can be considered exploratory that may warrant scrutiny with future studies. Notably, there was an exaggerated decrease in convex disc height in the -M group at the post-op timepoint (Fig. 5C), differing by 1.2 mm (*p* = 0.25) and 1.0 mm (*p* = 0.342) from the NM and + M groups, respectively, though not reaching statistical significance due to wide variability and small sample size.

**Fig. 6:**
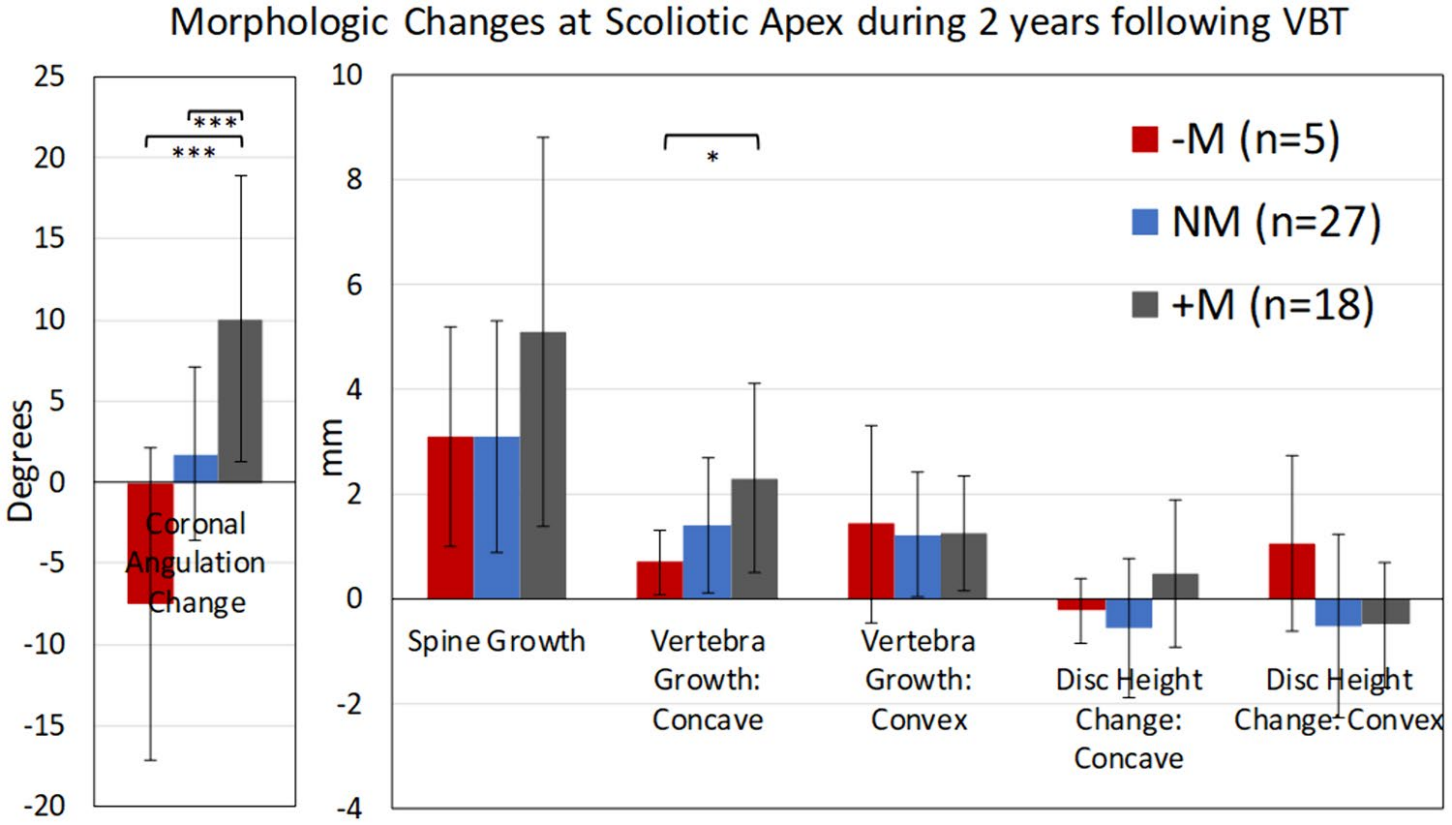
Morphologic changes of the curve apical 3 vertebrae and 2 discs following VBT: change among growth modulation groups between post-op and 2-year timepoints. “ + M” = High modulator, “NM” = Neutral modulator, “-M” = Poor modulator. Mean ± SD. Kruskal–Wallis Test with Dunn Post-hoc pairwise comparison (adjusted). *p < 0.05, **p < 0.01, ***p < 0.001. The NM group’s apex had grown/lengthened 3.1 mm, with mean concave vertebra height increasing 1.4 mm while the convex increased 1.2 mm, without large changes in the disc heights during this time. The -M group experienced comparable increases in spine length (3.1 mm), with asymmetry of concave and convex vertebra growth (0.7 mm vs. 1.4 mm). The + M group experienced 5.1 mm spine growth, with a similar amount of convex growth (1.3 mm) but a greater increase in concave growth of 2.3 mm

Looking at the morphologic change occurring between timepoints stratified by group also yielded notable results (Fig. 6). The NM group’s apex had grown/lengthened 3.1 mm, with mean concave vertebra height increasing by 1.4 mm while the convex increased 1.2 mm, without large changes in the disc heights during this time. The -M group experienced comparable increases in spine length (3.1 mm), with asymmetry of concave and convex vertebra growth (0.7 mm vs. 1.4 mm). The + M group experienced 5.1 mm spine growth, with a similar amount of convex growth (1.3 mm) but a greater increase in concave growth (2.3 mm). There was a significant difference between + M and -M mean concave vertebral height growth over this period (2.3 mm vs. 0.7 mm; *p* = 0.039), while convex growth was similar among all groups (*p* = 0.91).

## Discussion

The specific mechanisms that govern continued correction of a scoliotic curve following vertebral body tethering remain understudied. The wide variability of this response remains a significant barrier to achieving predictable outcomes with VBT. Our aim was to identify morphologic vertebra and disc changes that accompany the growth modulation response in the 2-years following VBT placement using a 3D analysis with the largest such dataset to date.

Our study confirmed a varied GM response among the cohort, with concave vertebral body growth and spine growth positively correlating with increasing GM magnitude, while convex disc height increase negatively correlated with GM. This study also seems to outline the mechanisms for immediate and gradual correction following VBT: immediate correction being chiefly attributable to convex disc height loss, and GM being chiefly attributable to asymmetric vertebral body growth over time, driven primarily by the magnitude of concave vertebra growth.

Growth modulation following VBT has been a focus of research in recent years due to its clinical importance in determining successful VBT outcomes. Takahashi and Newton, *et.al.* used screw angulation in 23 patients to determine the rate of angular correction following VBT [11], finding that GM occurs primarily within the first two years following VBT, up to three years in the most skeletally immature (Sanders 2) patients. GM response did correlate with patient height increases in their study, but the morphologic spine changes that accompany the GM response were not investigated. McDonald, *et.al.* were the first to study these morphologic changes in cases of successful GM, albeit using 2D measurements [12]. Their data formed the basis for future hypotheses on the GM response in humans, demonstrating a reduction of convex disc height as well as differential vertebra height growth with 2.0 mm concave vs. 1.5 mm convex growth in two years. A recent series by Farivar, et. al. challenges the conclusion that asymmetric vertebral growth drives GM, though their methodology did not account for residual deformity and remains unvalidated [13]. Our results support the conclusions of McDonald, *et.al.*, as our cohort overall demonstrated asymmetric vertebra growth with a mean of 1.7 mm concave and a mean of 1.3 mm convex growth.

Due to the known limitations of a 2D approach, including poor reproducibility and difficulty accounting for sagittal vertebra tilt and axial rotation [14], 3D analysis has been recently explored. Newton, et.al. piloted the methodology of 3D reconstructions of biplanar radiographs analyzed by 3D SAMS software to study 14 patients [15]. They found a 2-year growth differential of 1.5 mm in their patients who experienced GM phenomena. This study by Newton, et.al. first demonstrated the utility of a 3D approach, though conclusions about differences among groups were limited due to the small sample size.

Our correlative data demonstrate three morphologic changes associated with the GM phenomena (in order of importance): concave vertebra growth (*r* = 0.57), convex disc height decrease (*r* = − 0.42), and 3D spine length increase (*r* = 0.36). Unlike the McDonald, *et.al.* cohort, which was selected to contain successful GM cases only, our cohort experienced varied GM magnitude, and therefore the average growth differential of the cohort was more modest. However, this approach allowed distinction between GM successes and failures. Our GM group analysis, which focused on differences in apical changes among groups who varied in their GM response, adds insight into which important morphologic changes occur in each group. In our High Modulator “ + M” group, asymmetric vertebrae growth was exaggerated at 2.3 mm concave vs. 1.3 mm convex, while there was no observable differential in Neutral Modulator patients (1.4 mm vs. 1.2 mm) and a trend reversal in the –M patients (0.7 mm vs. 1.4 mm). Expected vertebral growth is estimated to be around 1 mm per year in adolescents [16], which indicates that concave vertebra growth resumes at near-physiologic rates in select patients following tether placement, while convex vertebra growth is partially (but not fully) attenuated by the tether in most individuals.

The inclusion of more patients and more varied GM responses adds depth to our existing understanding of the GM response and prompts future study. Though only five patients were represented, the data of the -M group of Poor Modulators imply some interesting possibilities. First, they are the only group that experienced an increase in convex apical disc height over the course of the study. Newton, et.al. previously suggested that the total length of the convexity remains constant if the convex tether remains intact [15]. Because the convex vertebra height seems to uniformly increase despite tethering, there was a corresponding decrease in convex disc height in their cohort. When disc height is permitted to rebound (as was seen in select patients in this study, presumably due to tether discontinuity), one can expect deteriorated ability for modulation or curve recurrence. Of particular interest is the finding in Fig. 5C where the –M convex disc heights were relatively more decreased than other groups at post-op, perhaps indicating increased tether tension. That height loss was not maintained, as convex disc height rebounded by 2-years. Most experts agree that increasing tether tension positively influences initial deformity correction and eventual growth modulation. However, these findings raise the question as to whether excessive tension beyond a threshold that induces GM may predispose to tether rupture and perpetuate the exact outcome we are meaning to avoid. Additional analysis focused on risk factors for tether rupture and the effect on GM are warranted.

Understanding morphologic changes associated with GM may have implications beyond VBT into non-operative, non-fusion methods of scoliosis management. Multiple studies now indicate that long-term improvement of the Cobb angle can be achieved through altering biomechanical forces with casting or bracing growing patients [17–19]. We suggest a possible mechanism for this coronal angulation improvement: growth modulation through a change in vertebral morphology. A segmental analysis of GM that considers technical factors such as tension or segmental morphologic change induced by VBT, in addition to patient-specific factors, is a critical next step to better understand how to influence the desirable morphologic changes demonstrated herein.

There are limitations to this study inherent to the study design and available data. Complete data was unavailable for a majority of eligible patients (88/138) leading to their exclusion. While regrettable, we do not feel this missing data confounds the results of the VBT procedure and thus the conclusions herein remain valid. We focused our study on the first two years following VBT, as prior studies have indicated this period as being most influential and critical in the growth modulation response. Of course, the results of VBT must be durable over the long-term, and it is possible that tether rupture or degenerative changes occurring in the medium to long-term may alter VBT results. However, the 2-year outcomes used in this study allow for a more narrowed focus on the GM response when it is most pronounced. We did not evaluate interval timepoints prior to two years due to resource and radiograph availability. This makes determining changes in growth rate impossible and can lead to underestimation of tether rupture rates. The time interval between pre-op and post-op was also sizable (mean 86.4 days), which likely limits the precision of some measurements. For instance, the curve may have progressed from pre-op to surgery; or there could have been some early GM occurring after tethering but prior to post-op evaluation (42.2 days). This is a likely explanation for why we observed a small statistical increase in spine length and concave vertebra height from pre-op to post-op timepoints. We also lacked hand radiographs for Sanders scoring for most patients in this cohort, as these were early VBT procedures and Sanders scoring was not routinely utilized [17, 18]. Fortunately, the recently published proximal femoral maturity index [7] is a suitable alternative, being both reliable and highly correlated with Sanders scoring and peak growth. PFMI is graded 0–6 (7 grades) with grade 3 indicating Peak Height Velocity and grade 6 indicating growth cessation. Fourteen of 18 (78%) of our + M group were PFMI 2–3 (immediately prior to, or within peak growth phase), which is consistent with prior assertions that GM occurs predominantly in patients with Sanders 2–3 skeletal maturity. Hand radiographs are now routinely obtained and future studies will include multiple benchmarks for skeletal maturity.

In conclusion, we conducted a thorough analysis of the morphologic changes of vertebra and discs that accompany spine growth modulation in the two years following VBT, leveraging the accuracy of 3D reconstructions with the largest such dataset. Acute correction of the curve occurred with convex tether placement and loss of convex disc heights. Successful GM from first-post-op to 2 years was characterized by near-physiologic concave vertebrae growth, which outpaced the stunted, but consistent, convex vertebrae growth. A poor GM response is characterized by continued attenuation of concave vertebra growth, and in some cases, tether rupture and rebound of the prior convex disc height loss. The precise role of tether rupture and other factors, such as tether tension or patient-specific factors, remain to be elucidated.

## Data Availability

All data supporting the findings of this study are not openly available due to sensitivity, however they are available to collaborators within the Pediatric Spine Study Group with appropriate Data Use Agreements in place.
